# Essential Oil Composition and Internal Transcribed Spacer (ITS) Sequence Variability of Four South-Croatian *Satureja* Species (Lamiaceae)

**DOI:** 10.3390/molecules14030925

**Published:** 2009-02-27

**Authors:** Nada Bezić, Ivica Šamanić, Valerija Dunkić, Višnja Besendorfer, Jasna Puizina

**Affiliations:** 1 University of Split, Faculty of Science, Department of Biology, Teslina 12, 21000 Split, Croatia; 2 University of Zagreb, Faculty of Science, Division of Biology, Department of Molecular Biology, Horvatovac 102A, Zagreb 10000, Croatia

**Keywords:** *Satureja montana*, *Satureja cuneifolia*, *Satureja subspicata*, *Satureja visianii*, Internal transcribed spacer (ITS), Chemotaxonomy, Molecular taxonomy.

## Abstract

The purpose of this study was to compare the essential oil profiles of four South-Croatian *Satureja* species, as determined by GC/FID and GC/MS, with their DNA sequences for an internal transcribed spacer (ITS1-5.8S-ITS2) of the nuclear ribosomal DNA. A phylogenetic analysis showed that *S. montana* and *S. cuneifolia*, characterized by a similar essential oil composition, rich in the monoterpene hydrocarbon carvacrol, clustered together with high and moderate bootstrap support. On the contrary, *S. subspicata* and *S. visianii*, characterized by quite unique essential oil compositions, clustered together with the moderate bootstrap support. All four Croatian *Satureja* species clustered in one clade, separately from Macaronesian *S. hortensis*, although it had essential oil composition similar to that of *S. montana* and *S. cuneifolia*. This is the first report on the comparison between the phytochemical and DNA sequence data in *Satureja* species and useful contribution to the better understanding of interspecies relationships in this genus.

## Introduction

The genus *Satureja* is comprised of some 200 species of often aromatic herbs and shrubs widely distributed in the Mediterranean Area, Asia and boreal America. In the Croatian flora nine species of genus *Satureja* have been reported, four of which are distributed in the Mediterranean region: *Satureja montana* L., *S. cuneifolia* Ten., *S. subspicata* Vis. and an endemic one *S. visianii* Šilić [1,2]. All of them are annual or perennial semi-bushy plants that inhabit arid, sunny, stony and rocky habitats along the Adriatic coast. Winter savory (*S. montana*) and wild savory (*S. cuneifolia*) are the most prevailing in this part of Croatia. 

Due to presence of secondary metabolites such as flavonoids, steroids, essential oils, and tannins, *Satureja* species have been known for their healing properties for a long time and have been used as traditional folk remedies to treat various ailments such as cramps, muscle pains, nausea, indigestion, diarrhea and infectious diseases. Their antimicrobial activity against a wide spectrum of multidrug-resistant pathogens has been confirmed [3,4,5,6,7,8]. In addition, recent experiments have confirmed their strong antioxidative and antiproliferative effects on human tumor lines [9] as well as anti-inflammatory and anti-nonciceptive effects on rats [10] and oils represent sources of natural larvicidal substances [11]. The antiviral activity of savory’s essential oils against HIV has been documented [12]. Savory’s essential oils in small quantities strongly inhibited the growth of food borne pathogens and several reports indicated that they may provide useful alternative to conventional synthetic antimicrobial additives in foods [13,14,15]. The antibacterial and antiviral mechanism of action of the major compound in savories essential oils, lipophilic terpenes (volatile mono- and sesquiterpenes), is most likely based on their solubility in biomembranes [16]. At their higher concentration, they influence the environment of membrane proteins (ion channels, transporters, receptors) and thus change their conformation and bioactivity. The mechanism of action of savory essential oils against cell membranes and walls of bacteria was confirmed by measurements of the intracellular pH, ATP concentration and the electronic microscopy observations of the bacterial cells treated with essential oils [17,18]. Essential oils are produced and secreted in glands on the leaf surface and flowers and it is a specific anatomical characteristic of all aromatic species of the family Lamiaceae. Representatives of the genus *Satureja* typically bear glandular hairs that secrete essential oils. Volatile monoterpenes, a typical constituent of their essential oils and of other members of the subfamily Nepetoideae, are produced in the disc cells with leucoplasts and ultrastructural changes during this process were documented [19,20,21,22]. The anatomy of the glandular apparatus (glandular cells and adjoined epidermal cells) as well as the essential oil content were proposed to be elements for the recognition of separate *Satureja* group and thus helpful in solving very complex taxonomy problems of the *Satureja* group [23,24]. 

In contrast to numerous papers reporting about chemical composition of *Satureja* secondary metabolites, particularly essential oils, as well as their antimicrobial activity and other possible applications in fitotherapy, we observed a relative paucity of data on molecular taxonomy/phylogeny in this group. The only available data originate from [25] who revised the classical systematics of the subfamily Nepetoideae using the chloroplast *rbcL* gene sequences as a molecular marker. They supported the proposition of Cantino et al. [26] who placed the genus *Satureja* in the tribe Menthae, in contrast to classical systematics which placed this genus within the tribe Saturejeae. To our knowledge, there are no published reports on intrageneric molecular phylogenetic or taxonomic studies in the genus *Satureja*. 

The objectives of the present study were: *i*) to determine and compare the essential oil composition of four wild-grown *Satureja* species from the Mediterranean part of Croatia (*S. montana*, *S. cuneifolia,* *S. subspicata and* *S. visianii* *)* *ii*) to determine their genetic relatedness by using sequence data for the internal transcribed spacer (ITS1-5.8S-ITS2) region of nuclear ribosomal DNA (nrDNA) *iii*) to discuss the comparison between the phytochemical and DNA sequence data taking into account also morphology, ecology and geographic distribution of the investigated *Satureja* species.

## Results and Discussion

### Essential oil composition

Essential oils were obtained by hydrodistillation of the aerial parts of the plant in flowering period and were then analyzed by GC/FID and GC/MS. The specific identified compounds and their percentages are given in Table 1. Twenty seven components, representing 92.85% of the oil, were identified in *S. montana*, 26 compounds, representing 89.8% of the oil in *S. cuneifolia*, 29 compounds, representing 93.2% of the oil in *S. subspicata* and 46 compounds was identified in *S. visianii*, which represents 92.2% of the oil. The essential oils isolated from flowering vegetative cycle were obtained in yields: 2.8% in *S. montana*, 2.6% in *S. cuneifolia*, 2.0 % in *S. subspicata* and 2.4% in *S. visianii*. Therefore, these four species can be assigned to oil-rich species of the Lamiaceae, in which the oils present a large diversity of volatile constituents [27]. Our results showed that the major compounds in the essential oil of *S. montana* were phenolic monoterpene carvacrol (13.7%), *p*-cymene (11.8%) and *γ*-terpinene (10.6%) then acyclic monoterpene alcohol linalool (4.6%) and limonene (9.5%) (Table 1). Similar results were obtained for S*. cuneifolia*: carvacrol (17.7%), *γ*-terpinene (14.8%), *p*-cymene (9.8 %), linalool (6.6%) and limonene (6.2%) (Table 1). Interestingly, the major compounds in the essential oil of S*. subspicata* were monoterpenic hydrocarbons *α*-pinene (24.2%), limonene (7.1%) and α-terpinene (6.2%), whereas the oil of *S. visianii* contained bicyclic monoterpene camphor (18.7%) as the major compound, α-thujene (10.9%), α-pinene (5.8%) and limonene (5.1%) (Table1).

### Essential oils variability

Monoterpene hydrocarbons and their derivatives dominate in the chemical composition of essential oils of all savory species analyzed so far, while sesquiterpene compounds are present in small quantities. Generally, the essential oils of the investigated savories show a large interspecies variability containing different percentages and types of common compounds such as: carvacrol, thymol, β-caryophyllene, γ-terpinene, *p*-cymene, linalool, and others [28,29,30,31,32,3]. 

Results obtained in our work are comparable to other published studies. The percentage of carvacrol in different samples of *S. montana* varied from 84% for some samples collected from the central part of Dalmatia [28], 57% in some Italian winter savories [33] up to 5.3% in some samples reported by [31] where thymol has been a dominant compound (45%). It has been shown that the production of phenolic compounds is stimulated by hot and dry conditions of the environment. Yield of carvacrol varied during ontogenesis and peaked at flowering time, which coincides with maximal summer temperatures.

Similarly to our results [29] found that among 19 samples of *S. cuneifolia* from Turkey, eleven samples were found to be rich in carvacrol (26-72%) while in eight samples thymol (22-58%). Based on our results and above mentioned published data it is obvious that both savory species *S. montana* and *S. cuneifolia* have carvacrol and/or thymol as major constituents of their essential oils. Carvacrol and thymol are isomeric compounds containing a (1-methylethyl) phenol group in their structures and the only difference of these two phenol derivatives is the position of hydroxyl group on their phenol ring and they both have a very close biosynthetic relationship with their precursors, γ-terpinene and *p-*cymene. Akgul *et al*. [34] proposed that due to phenolic constituents, carvacrol and thymol, of different *Satureja* species from Turkey, which dominated in their essential oils, these species could be assigned as genuine carvacrol/thymol chemotype. A similar conclusion reached Biavati *et al*. [35] for some Italian savory species. Based on our results of essential oil analysis of two Croatian *Satureja* species (*S. montana and S. cuneifolia*) we support such a conclusion.

A high concentration of α-pinene as found in *S. subspicata* in this work was a quite surprising result since α-pinene is not characteristic of other *Satureja* species, but it is specific to woody plants such as the genus *Pinus* [36] and some *Salvia* species. For sage, the possibility of nonenzymatic conversion to α-pinene from other compounds was excluded, indicating direct cycling from geranyl pyrophosphate to α-pinene [37]. It is very likely that this compound in savory is synthesized in a similar way as in sage. Based on this quite unique essential oil composition we assigned *S. subspicata* as having the α-pinene chemotype 

Another interesting result was a relatively high concentration of camphor (9.4%) in essential oil of *S. visianii*. The only *Satureja* species with camphor (9.4%) as the main component of essential oil identified so far was *S. isophylla* from Iran [27]. Although previous studies showed that the major compound in other species of *Satureja* were thymol and carvacrol, *S. visianii* contained those compounds at not more than 2.6%. Based on this finding, it is evident that *S. visianii* possess a quite unique essential oil composition among all other *Satureja* species, investigated so far, and we classified it as having the camphor chemotype. It is interesting that γ-terpinene (precursor for biosynthesis of carvacrol and thymol and other simple isomeric monoterpenic phenols) was not found in *S. subspicata* and *S. visianii*. It is worth mentioning that all the oils of our four savory plants analyzed in this paper were characterized by an alkali content of limonene (Table 1), which is a natural monoterpene with chemotherapeutic activity [38].

Therefore we concluded that, based on essential oil composition, the four investigated *Satureja* species could be grouped into three chemotypes: *i*) phenol chemotype which ends up with carvacrol or thymol as major products (*S. montana and S. cuneifolia*), *ii*) α – pinene chemotype (*S. subspicata)* and *iii*) camphor chemotype (*S. visianii*).

In order to see whether such a grouping is also supported by DNA sequence data we performed molecular analysis and compared the two sets of data. 

**Table 1:** Phytochemical composition (%) of essential oils of *Satureja montana*, *S. cuneifolia*, *S. subspicata* and *S. visianii.*

No.	Component	RI	*S. montana* year (yield %) 2007 (2.8)	*S. cuneifolia* year (yield %) 2007 (2.6)	*S. subspicata* year (yield %) 2007 (2.0)	*S. visianii* year (yield %) 2007 (2.4)
1.	α-Thujene	924	-	-	-	10.9
2.	α-Pinene	935	0.9	-	24.2	5.8
3.	Camphene	947	-	-	-	1.7
4.	Verbenene	961	-	-		0.4
5.	Sabinene	971	-	-	1.7	1.4
6.	1-Octene-3-ol	974	1.1	1.4	1.8	1.3
7.	Myrcene	988	4.3	-	2.1	1.6
8	Linalool oxide	991	-	0.3	1.3	0.2
9	α-Terpinene	1016	-	-	6.2	-
10.	*p*-Cymene	1021	11.8	9.8	t	0.3
11.	Limonene	1028	9.5	6.2	7.1	5.1
12.	(Z)-β-Ocimene	1032	-	-	4.8	1.7
13.	γ-Terpinene	1057	10.6	14.8	-	1.6
14.	n-Octanol	1063	-	2.4	1.4	2.1
15.	Sabinene hydrate	1065	-	-	1.2	0.3
16.	Methyl benzoate	1088	-	-		t
17.	Linalool	1097	4.6	6.6	1.2	2.3
18.	*allo-*Ocimene	1128	0.6	2.1	1.7	0.4
19.	Camphor	1143	1.2	-	-	18.7
20.	Camphene hydrate	1145	-	-	-	0.5
21.	Menthone	1148	-	-	-	0.2
22.	Isoborneol	1155	1.1	0.6	1.1	0.4
23.	Borneol	1165	5.8	4.2	1.8	-
24.	Terpinen-4-ol	1174	2.7	2.3	1.3	1.8
25.	α -Terpineol	1186	1.3	0.8	1.8	5.1
26.	Myrtenol	1194	-	2.1	1.3	0.8
27.	Nerol	1227	0.7	-	-	1.4
28.	Geraniol	1249	3.6	2.9	2.4	3.6
29.	Linalyl acetate	1261	3.7	2.2	2.2	3.8
30.	Thujanol acetate	1281	-	-	-	0.3
31.	Thymol	1290	1.9	2.3	3.9	2.3
32.	Carvacrol	1298	13.7	17.7	2.7	0.6
33.	α -Terpinyl acetate	1316	1.9	-	1.5	0.3
34.	Neryl acetate	1359	-	2.6	2.6	2.0
35.	α -Copaene	1374	-	0.9	4.8	1.4
36.	Geranyl acetate	1379	3.7	3.2	2.4	5.4
37.	β-Bourbonene	1387	0.9	0.4	1.6	0.4
38.	Aromadendrene	1439	0.3	-		0.1
39.	α -Humulene	1452	0.8	-	-	1.0
40.	(E)-β -Farnesene	1454	-	-	-	0.4
41.	β -Caryophyllene	1467	1.2	0.3	-	0.5
42.	Viridiflorene	1496	-	0.3	2.3	0.2
43.	δ-Cadinene	1522	2.3	2.9	4.8	0.1
44.	Spathulenol	1578	1.0	0.3	-	0.9
45.	Caryophyllene oxide	1582	1.6	0.2	-	1.6
46.	α -Eudesmol	1652	-	-	-	0.6
47.	Elemol acetate		-	-	-	0.2
48.	Heptadecane		-	-	-	0.5
	Total:		92.8	89.8	93.2	92.2

RI, retention indices, RI CP Sil 8 CB; t, trace<0.1%.

### Internal transcribed spacer (ITS) sequence analysis

For the purpose of assessment of the intrageneric relationship between the four South-Croatian *Satureja* species we amplified by PCR the entire internal transcribed spacer (ITS1-5.8S-ITS2). Although routinely used in our lab, PCR amplification with genomic DNA isolated by CTAB buffer gave no PCR amplification. Successful PCR amplification was only obtained after repetitious rounds of purifications with phenol-chlorophorm-isoamyl alcohol (25:24:1) and DNA precipitations with isopropanol since the investigated savory species are oil-rich and also contain high amount of phenolic compounds. 

As direct sequencing of the PCR products gave rather poor results, we cloned PCR products and sequenced the clones. Two to four clones of *S. montana* (EU823287, consensus sequence), *S. visianii* (EU823289, consensus sequence) and *S. cuneifolia* (EU823290, consensus sequence), and one clone of *S. subspicata* (EU823288) were obtained. For the purpose of comparison with our sequence data, we have taken the corresponding sequence of *S. hortensis* (AY227143) originating from the Macaronesian Islands and the only ITS sequence of *Satureja* species deposited in GenBank. As an outgroup we used ITS sequences from O*riganum vulgare* (AY506647) also taken from GenBank.

**Figure 1:**
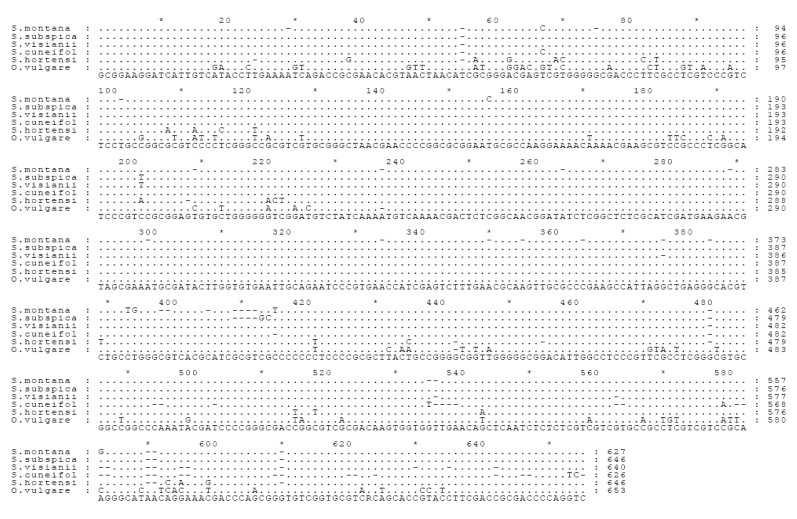
The alignment of ITS consensus sequences of *S. montana* (EU823287), *S. subspicata* (EU823288), *S. visianii* (EU823289), *S. cuneifolia* (EU823290). Sequences for *S. hortensis* (AY227143) and *O. vulgare* (AY506647) have been taken from the GeneBank

**Figure 2:**
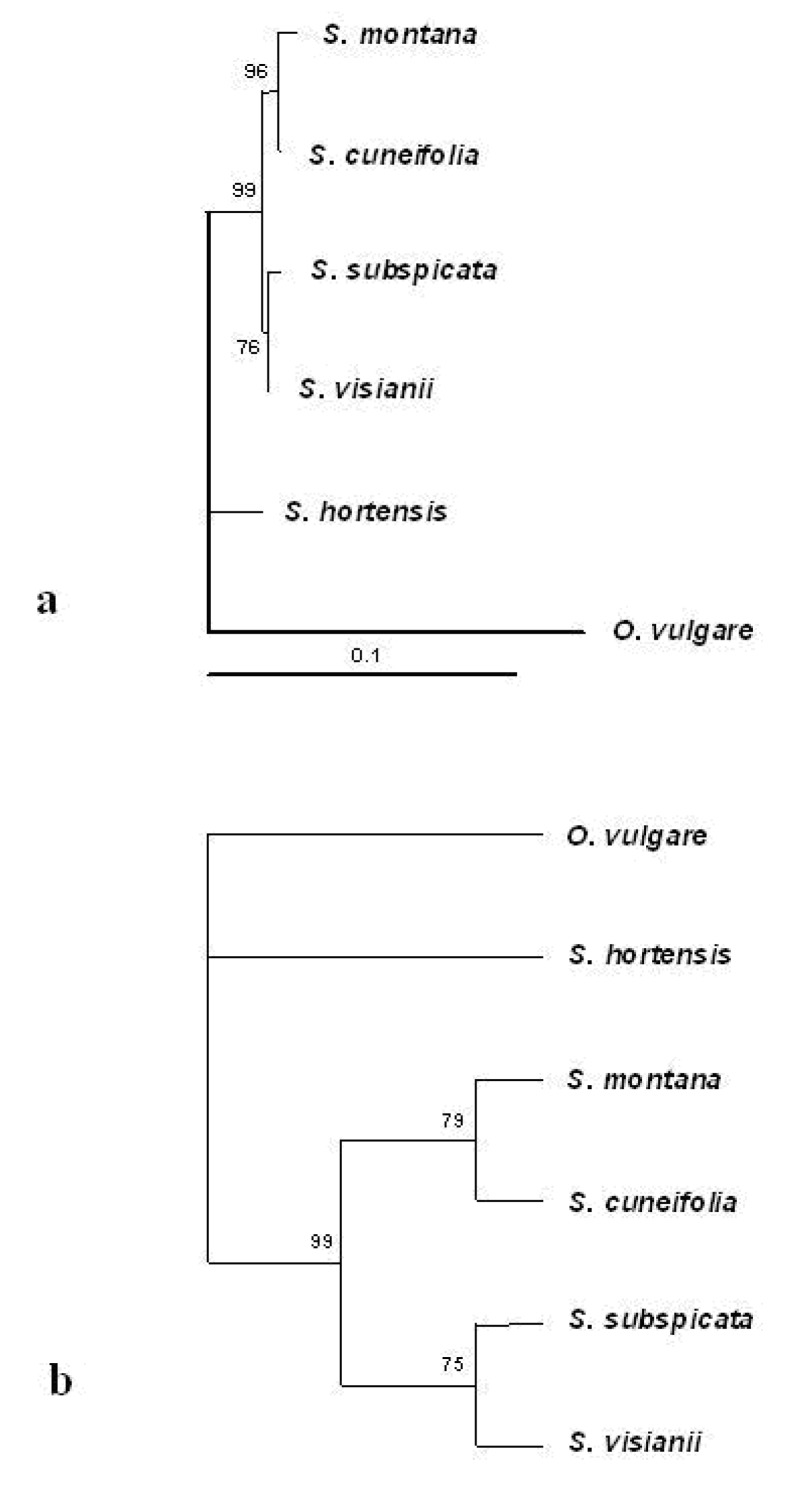
Phylogenenetic relationship of *Satureja* species based on ITS sequences.

The length of ITS consensus sequences of *S. montana*, *S. visianii*, *S. subspicata* and *S. cuneifolia* varied from 627-646 bp and they showed 92-98% of similarity (Figure 1). Both phenetic trees obtained either by Neighbour-joining or Maximum parsimony clearly showed that all investigated Croatian species belong to one phylogenetic group of *Satureja* species, with Macaronesian *S. hortensis* having distant position (Figure 2). Moreover, both phenetic trees showed that *S. montana* and *S. cuneifolia* cluster together with high and moderate bootstrap support (96 and 79, respectively) as well as *S. subspicata* and *S. visianii* that cluster together with the moderate bootstrap support (76 and 75, respectively). 

The analysis of DNA sequences based on PCR amplification and sequencing of the selected molecular markers became frequent and widely accepted molecular technique and powerful approach for reconstructing the phylogeny and taxonomy in plants. In contrast to animals where the major molecular marker used for phylogenetic studies is a short DNA sequence in the mitochondrial cytochrome c oxidase 1 (CO1) gene [39], plant mitochondrial DNA sequences have only rarely been used as a source of phylogenetic markers because of their presumed slow rate of nucleotide substitutions. Bakker *et al*. [40] and some other studies confirmed that plant mitochondrial sequences do contain levels of variation suitable for species-level discrimination, however, the internal transcribed spacer (ITS) region of the nuclear ribosomal cistron (18S-5.8S-26S) remains the most commonly sequenced locus for plant molecular systematic investigations [see for review 41, 42]. Although some authors are of opinion that due to its complex and unpredictable evolutionary behaviour it has reduced utility for phylogenetic studies [41], recently it has been proposed by Kress *et al*. [42] that the ITS region and additional plastid region *trn*H-*psb*A intergenic spacer are potentially usable DNA regions for applying in barcoding to flowering plants. 

Both Neighbour-joining and Maximum parsimony phenetic trees indicate that winter savory, *(S. montana)* and wild savory, *(S. cuneifolia)* were genetically rather close to each other, whereas the mountain savory, (*S. subspicata)* and an endemic savory species, S*. visianii* were genetically more distant.

ITS data agree well with morphological and geographical characters of the analyzed *Satureja* species: *S. montana* and *S. cuneifolia* are the most prevailing of all other *Satureja* species, in this part of Croatia. *S. montana* is widely distributed in entire Mediterranean Region as well as Central Asia. On contrary, *S. cunefolia* has clearly limited areal in Italy, Croatia, Bosnia and Herzegovina, Monte Negro and Albania, but on the north-east it continues on species *S. obovata* Lag. and on the east on *S. pilosa* Velen. Winter and wild savory share many morphological similarities in the flower and leaves morphology. However, typical feature of the wild savory is its stem densely covered by hairs. In contrast to these two savory species*, Satureja subspicata*, is a rare mountain savory, narrowly distributed on the Adriatic coast on open rocky Dinaride of Croatia. Interestingly, Šilić [1] reports absence of this savory species from Adriatic Islands. Outside Croatia *Satureja subspicata* has an area of distribution in Italy near Trieste, the Slovenian part of the Istrian Peninsula (south Notranjska), Bosnia and Hercegovina, Monte Negro and Macedonia, and northern Albania. The plant is perennial shrub sprouting every spring with new twings full of linear and leathery leaves and purple flowering during October. The endemic species *S. visianii* Šilić differs from all other species of the genus *Satureja* not only with regard to morphology of the flower, it has yellowish corolla with purple dots and stripes, but with regards to its geographical distribution as well - it grows only in a narrow zone of the Pelješac Peninsula. 

The ITS sequence of *Origanum vulgare* was used as outgroup. Phenetic trees were obtained by (a) Neighbor-joining and (b) Maximum parsimony analysis. Neighbor-joining was done according to Kimura′s distance matrix [46]. One phenetic tree was obtained by Maximum parsimony exhaustive search with a tree length of 109 with 555 monomorphic nucleotides out of 655 and 14 characters were parsimony-informative. Bootstrap values from 1,000 replicates are given on the branches in both phenetic trees. 

### The comparison between the phytochemical and molecular DNA data

The comparison between the molecular DNA data and chemoprofiles of the analyzed species showed rather complex situation. Although the phylogenetic tree based the ITS sequences of the Croatian *Satureja* species principally corrobated their division into the three chemotypes, the situation changed with the addition of the fifth *Satureja* species to the phylogenetic tree - Macaronesian *S. hortensis*. It was the only *Satureja* species whose ITS sequence was publicly available and deposited in the GenBank*.* The final phenetic analysis positioned *S. hortensis* separately and outside of the cluster of four Croatian *Satureja* species, although it had the essential oil of carvarcol chemotype that is quite similar to that of *S. montana* and *S. cuneifolia.* According to Güllüce *et al*. [3], the main constituents of *S. hortensis* essential oil were thymol (29%), carvacrol (26.5%), γ-terpinene (22.6%) and *p*-cymene (9.3%). 

Similar results reported Wink [16] when he compared the distribution and types of secondary metabolites with molecular phylogeny data in Lamiaceae: both sets of data confirmed the main subdivision into the two families: Lamioideae with iridoid glycosides and Nepetoideae with volatile monoterpenes as major classes of secondary metabolites. However, a few members of the Nepetoideae also produced iridoids. He concluded that the absence of iridoids in most but not all members of the Nepetoideae could be due to a change of genetic expression of the corresponding genes (their inactivation), which probably evolved earlier in evolution of Lamiaceae and are, very likely, common to all members of that family [16]. 

These two independent studies suggest that the distribution and type of secondary metabolites have some value for taxonomy but their occurrence more likely reflect adaptations to ecological conditions and particular life strategies embedded in a given phylogenetic framework and, therefore, as chemotaxonomic markers they have to be analyzed carefully and critically. 

## Conclusions

Although the majority of variations in essential oil composition among the analyzed *Satureja* species as found in this paper probably could be attributed to ecological conditions, life cycle and/or some other factors, we also observed some more profound differences which could, perhaps, indicate a different genetic basis. However, further analyses should be undertaken to explore and better understand this question. The comparison between the molecular and phytochemical sets of data of the four investigated *Satureja* species has not been reported before and therefore our results are a useful contribution to the taxonomy and better understanding of the interspecies relationships in the genus *Satureja*. 

## Experimental

### Plant material

Plant material of three *Satureja* species (*S. montana* L., *S. cuneifolia* Ten. and *S. subspicata* Vis.) was collected from the Kozjak Mountain (near the city of Split) and *S. visianii* species from the Pelješac Peninsula (near the city of Dubrovnik) in the summer (August) 2007. Voucher specimens are deposited in herbarium at Department of Biology, Faculty of Science, University of Split, Croatia [No.FNSMST 2006: 11, 12, 13 and 14].

### Isolation and GC-MS analysis of essential oils

Aeiral parts of plants were performed in a shady place at room temperature for 10 days. Plant tops during flowering were used for the analysis of essential oil composition. Dried aerial parts of plant material (100 g) were subjected to hydrodistillation for 3 h in a Clavenger type apparatus. The obtained essential oil was dried over anhydrous sodium sulphate and 1 µL was used for GC/FID and GC/MS measurements. The yield of the oil was for *S. montana* (2.8%), *S. cuneifolia* (2.6%), *S. subspicata* (2.0%) and *S. visianii* (2.4%).

GC/FID analyses were performed on Varian 3900 gas chromatograph equipped with a flame ionization detector (FID) and a CP Sil 8 CB capillary column (50 m x 0.25 mm, film thickness 0.12 μm) with a 5%-phenyl-95%-dimethylpolysiloxane as the stationary phase. Hydrogen was used as the carrier gas flow rate 1.2 mL/min; the injection volume 1 μL whit a split ratio 1:10, GC oven temperature was kept at 50^o^C for 5 min, and programmed to 250 °C at a rate of 5 °C/min. 

GC/MS analyses were carried out on a Varian Saturn 2000 system equipped with a CP Sil 8 CB capillary column; with similar temperature programmed as in GC, transfer line temperature 250°C, carrier gas helium with a linear velocity of 31.5 cm/s, split ratio 1/60, ionization energy 70 eV, ion source temperature 280 °C, mass range 40 – 600 mass units.

The individual peaks were identified by comparison of their retention indices of *n*-alkanes to those of authentic samples and literature [43], as well as by comparing their mass spectra with the Wiley 6.0 library (Wiley, New York) and NIST/02 mass spectral database. The percentage composition of the samples was computed from the GC peak areas using the normalization method.

### DNA isolation, PCR amplification, cloning and sequencing

Since the DNA obtained by standard methods for plant genomic DNA isolation based on CTAB buffer gave no PCR amplification, we isolated DNA using DNeasy Plant Mini Kit (Quiagen) followed by an additional step of purifying with phenol:chlorophorm:isoamylalcohol (25:24:1) and DNA precipitation with isopropanol. 

The ITS region was amplified by primers ITS-1 (5’GTTTCCGTAGGTGAACCTGC3’) and ITS-4 (5’TCCTCCGCTTATTGATATGC3’). PCR amplification was carried out in a 20 μL reaction containing 5-10 ng of DNA, 0.5 μM of each primer, 200 μM dNTPs 1.5 mM MgCl_2_ , 1x Taq buffer + (NH_4_)_2_SO_4_ – MgCl_2_ (Invitrogen) and 2 U Taq DNA polymerase (Invitrogen). After initial denaturation at 94 °C for 4 min, the amplification was carried out in 35 cycles consisting of denaturation at 94 °C, 15 sec, annealing at 55 °C, 30 sec, and primer extension at 72 °C 1 min, with a final extension step at 72 °C for 7 min. Amplified fragments were separated on 1 % (w/v) agarose gel.

For cloning purposes, the amplified DNA fragments were gel-purified using a Jetquick - Gel Extraction Spin Kit (Genomed) and cloned into the pCR 2.1.plasmid (TOPO-TA Cloning Kit, Invitrogen) according to manufacturer’s instructions. Chemically competent *Escherichia coli* TOP10’ cells (Invitrogen) were transformed and plasmid extractions from recombinant clones were performed using a Plasmid Mini Kit (Quiagen). Dideoxy chain-terminating sequencing reactions were carried out by DNA-servis at Institute “Rudjer Bošković” (Zagreb, Croatia). 

### Sequence alignment and phenetic analysis

ITS sequences of four *Saturjea* species were subjected to a similarity search against the nonredundant nucleotide sequence database using the NCBI (National Centre for Biotechnology Information) BLASTN network service. Sequence alignments were performed by using the ClustalX program refined manually using BioEdit [44] and visualized by GenDoc. To obtain phenetic tree Neighbour-joining method and Maximum parsimony analysis were performed using PAUP, version 4.0 beta [45].

## References

[1] Šilić Č. (1979). Monographie der Gattungen Satureja L., Calamimtha Miller, Micromeria Bentham, Acinos Miller und Clinopodium L. Der Flora Jugoslawiens.

[2] Pedersen J. (2000). Distribution and taxonomic implications of some phenolics in the family Lamiaceae determined by ESR spectroscopy. Biochem. Syst. Ecol..

[3] Güllüce M., Sökmen M., Daferera D., Ağar G., Ozkan H., Kartal N., Polissiou M., Sökmen A., Sahin F. (2003). In vitro antibacterial, antifungal, and antioxidant activities of the essential oil and methanol extracts of herbal parts and callus cultures of *Satureja hortensis* L. J. Agric. Food Chem..

[4] Gören A., Topçu G., Bilsel G., Bilsel M., Wilkinson J., Cavanagh H. (2004). Analysis of essential oil of *Satureja thymbra* by hydrodistillation, thermal desorber, and headspace GC/MS techniques and its antimicrobial activity. Nat. Prod. Res..

[5] Sahin F., Karaman I., Güllüce M., Oğütçü H., Sengül M., Adigüzel A., Oztürk S., Kotan R. (2003). Evaluation of antimicrobial activities of *Satureja hortensis* L. J. Ethnopharmacol..

[6] Skočibušić M., Bezić N. (2004 a). Chemical Composition and Antimicrobial Variability of *Satureja montana* L. Essential Oils Produced During Ontogenesis. J. Essent. Oil Res.

[7] Skočibušić M., Bezić N. (2004 b). Phytochemical analysis and in vitro antimicrobial activity of two *Satureja* species essential oils. Phytotherapy Res..

[8] Skočibušić M., Bezić N., Dunkić V. (2006). Phytochemical composition and antimicrobial activities of the essential oils from *Satureja subspicata* Vis. growing in Croatia. Food Chem..

[9] Cetojević-Simin D., Canadanović-Brunet J., Bogdanović G., Cetković G., Tumbas V., Djilas S. (2004). Antioxidative and antiproliferative effects of *Satureja montana* L. extracts. JBUON..

[10] Amanlou M., Dadkhah F., Salehnia A., Farsam H., Dehpour A. (2005). An anti-inflammatory and anti-nociceptive effects of hydroalcoholic extract of *Satureja khuzistanica* Jamzad extract. J. Pharm. Sci..

[11] Michaelakis A., Theotokatos S.A., Koliopoulos G., Chorianopoulos N.G. (2007). Essential OIls of *Satureja* species: Insecticidal Effect on *Culex pipiens* Larvae (Diptera: Culicidae). Molecules.

[12] Yamasaki K., Nakano M., Kawahata T., Mori H., Otake T., Ueba N., Oishi I., Inami R., Yamane M., Nakamura M., Murata H., Nakanishi T. (1998). Anti-HIV-1 activity of herbs in Labiatae. Biol. Pharm. Bull..

[13] Smith-Palmer A., Stewart J., Fyfe L. (1998). Antimicrobial properties of plant essential oils and essences against five important food-borne pathogens. Lett. Appl. Microbiol..

[14] Razzaghi-Abyaneh M., Shams-Ghahfarokhi M., Yoshinari T., Rezaee M., Jaimand K., Nagasawa H., Sakuda S. (2008). Inhibitory effects of *Satureja hortensis* L. essential oil on growth and aflatoxin production by *Aspergillus parasiticus*. Int. J. Food Microbiol..

[15] Rota C., Carramiñana J., Burillo J., Herrera A. (2004). In vitro antimicrobial activity of essential oils from aromatic plants against selected foodborne pathogens. J. Food Prot..

[16] Wink M. (2003). Evolution of secondary metabolites from an ecological and molecular phylogenetic perspective. Phytochemistry.

[17] Oussalah M., Caillet S., Lacroix M. (2006). Mechanism of action of Spanish oregano, Chinese cinnamon, and savory essential oils against cell membranes and walls of *Escherichia coli* O157:H7 and Listeria monocytogenes. J. Food Prot..

[18] Di Pasqua R., Betts G., Hoskins N., Edwards M., Ercolini D., Mauriello G. (2007). Membrane toxicity of antimicrobial compounds from essential oils. J. Agric. Food Chem..

[19] Turner G., Croteau R. (2004). Organization of monoterpene biosynthesis in *Mentha*. Immunocytochemical localizations of geranyl diphosphate synthase, limonene-6-hydroxylase, isopiperitenol dehydrogenase, and pulegone reductase. Plant Physiol..

[20] Turner G., Gershenzon J., Nielson E., Froehlich J., Croteau R. (1999). Limonene synthase, the enzyme responsible for monoterpene biosynthesis in peppermint, is localized to leucoplasts of oil gland secretory cells. Plant Physiol..

[21] Turner G., Gershenzon J., Croteau R. (2000). Development of peltate glandular trichomes of peppermint. Plant Physiol..

[22] Dunkić V., Bezić N., Ljubešić N., Bočina I. (2007). Glandular hair ultrastructure and essential oils in *Satureja subspicata* Vis. ssp. *subspicata* and ssp. *liburnica* Šilić. Acta Biol. Cracov. Series Bot..

[23] Hanlidou E., Kokkini S., Bosobalidis A.M., Bessiere J.M. (1990). Glandular trichomes and essential oil constituents of *Calamintha menthifolia* (Lamiaceae). Plant Syst. Evol..

[24] Bezić N., Dunkić V., Radonić A. (2001). Glandular apparatus structure and essential oil constituents of *Satureja cuneifolia* Ten. Acta Biol. Cracov. Ser. Bot..

[25] Kaufmann M., Wink M. (1994). Molecular systematics of the nepetoideae (family Labiatae): phylogenetic implications from rbcL gene sequences. Z. Naturforsch. C.

[26] Cantino P., Harley R., Wagstaff S., Harley R., Reynolds J. (1992). Genera of Labiatae status and classification. Advances in Labiatae Science.

[27] Sefidkon F., Jamazad Z. (2006). Essential oil analysis of Iranian *Satureja edmondi* and *S. isophylla*. Flavour Fragr. J..

[28] Kuštrak D., Kulftinec J., Blažević N., Maffei M. (1996). Comparison of the Essential Oil Composition of Two Subspecies of *Satureja montana*. J. Essent. Oil Res..

[29] Tümen G., Kirimer N., Ermin N., Başer K. (1998). The essential oil of *Satureja cuneifolia*. Planta Med..

[30] Azaz D., Demirci F., Satil F., Kürkçüoğlu M., Başer K. (2002). Antimicrobial Activity of Some *Satureja* Essential Oils. Z. Naturforsch..

[31] Radonić A., Milos M. (2003). Chemical composition and in vitro evaluation of antioxidant effect of free volatile compounds from *Satureja montana* L. Free Radic. Res..

[32] Baydar H., Sağdic O., Özkan G., Karadoğan T. (2004). Antibacterial activity and composition of essential oils from *Origanum*, *Thymbra* and *Satureja* species with commercial importance in Turkey. Food Contr..

[33] Angelini L., Carpanese G., Cioni P., Morelli I., Macchia M., Flamini G. (2003). Essential oils from Mediterranean Lamiaceae as weed germination inhibitors. J. Agric. Food Chem..

[34] Akgul A., Ozcan M., Chialva F., Monguzzi F. (1999). Essential oils of four Turkish wild-growing Labiatae herbs: *Salvia cryptantha* Montbr. Et Auch., *Satureja cuneifolia* Ten., *Thymbra spicata* L. and *Thymus cilicicus* Boiss. Et Bal. J. Essent. Oil Res..

[35] Biavati B., Ozcan M., Piccaglia R. (2004). Composition and antimicrobial properties of *Satureja cuneifolia* Ten. and *Thymbra sintenisii* Bornm. Et. Aznav. subsp. *isaurica* P.H. Davis essential oils. Ann. Microbiol..

[36] Bruneton J. (1995). Pharmacognosy Phytochemistry Medical Plants..

[37] Glambiel J., Croteau R. (1982). Biosynthesis of (±)-α-pinene and (-)-β-pinene from geranylpyrophsophate by a soluble enzyme system from sage (*SaMa officinalis*). J. Biol. Chem..

[38] Vigushin D., Poon G., Boddy A., English J., Halbert G., Pagonis C., Jarman M., Coombes R. (1998). Phase I and pharmacokinetic study of D-limonene in patients with advanced cancer. Cancer Research Campaign Phase I/II Clinical Trials Committee. Cancer Chem. Pharmacol..

[39] Hebert P., Ratnasingham S., deWaard J. (2003). Barcoding animal life: cytochrome c oxidase subunit 1 divergences among closely related species. Proc. R. Soc. B.

[40] Bakker F.T., Culham A., Pankhurst C.E., Gibby M. (2000). Mitochondrial and chloroplast DNA-based phylogeny of *Pelargonium* (Geraniaceae). Am. J. Bot..

[41] Alvarez I., Wendel J. (2003). Ribosomal ITS sequences and plant phylogenetic inference. Mol. Phylog. Evol..

[42] Kress W., Wurdack K., Zimmer E., Weigt L., Janzen D. (2005). Use of DNA barcodes to identify flowering plants. Proc. Nat. Acad. Sci. USA.

[43] Adams R.P. (2007). Identification of essential oil components by gas chromatography/ mass spectroscopy.

[44] Hall T. BioEdit v5.0.9.

[45] Swofford D. (1998). PAUP, Phylogenetic Analysis Using Parsimony (*and or Other Methods), Version 4.

[46] Kimura M. (1983). Rare variant alleles in the light of the neutral theory. Mol. Biol. Evol..

